# Establishment of the New Zealand white rabbit animal model of fatty keratopathy associated with corneal neovascularization

**DOI:** 10.1515/biol-2021-0111

**Published:** 2021-12-03

**Authors:** Yikui Gao, Cong Li, Xiaoyun Li, Minghong Zhang

**Affiliations:** Ophthalmology Department, The Eighth People’s Hospital of Qingdao, Qingdao 266000, China; Refraction Department, Qingdao Aier Eye Hospital, Qingdao 266400, China

**Keywords:** neovascularization, cornea, animal modeling, keratopathy, rabbit

## Abstract

The term fatty keratopathy is used to describe the phenomenon of fat deposition caused by corneal neovascularization, which will severely affect the eye’s beauty and vision. The purpose of this study was to establish a New Zealand white rabbit animal model of fatty keratopathy, that is, the establishment of an animal model of fatty keratopathy. The goal was achieved by the combination of a corneal neovascularization animal model and a hyperlipidemia animal model. Two groups were created according to the experimental sequence. The first group initially induced a corneal neovascularization pattern and later induced a hyperlipidemia pattern, and the second group followed the opposite sequence. The results of the two groups showed that all the significant crystalline deposits of the cornea were visible. So the animal models of fatty keratopathy were successfully established in both groups.

## 1 Introduction

The balanced and quiescent state of the cornea, like the transparent, vascular tissue of the eyes, is maintained by highly expressed factors that resist the growth of blood vessels and lymphatic vessels, as well as a small vascular growth factor, which actively retains the characteristics of the cornea’s avascular distribution, which is an essential condition for a clear and transparent cornea and the immune-privileged site [1,2,3,4]. Corneal transparency determines the degree of visual clarity and vision [5]. Through tears, aqueous humor, and the immune cells in blood circulation, surrounding tissues and plasma can interact with the corneal dynamically to maintain normal structure and function [6,7]. Among corneal diseases worldwide, neovascularization is one of the leading causes of hypoxia and even blindness. It is estimated that 4% of people in the United States suffer from corneal neovascularization, and this problem occurs in about 1.4 million people each year, of which 12% of patients suffer from hypoxia [8]. The mechanism of corneal neovascularization has not been fully clarified. After suffering, injuries such as hypoxia, inflammatory reactions, infections, allergies, toxins, degenerative diseases, and trauma, the balanced and quiescent state of the avascular cornea may be destroyed, thereby causing corneal neovascularization [9,10,11]. This is a normal natural physiological healing process, but if an improper local tissue response is generated in response to these stimuli, a chronic and continuous excessive angiogenesis response occurs, resulting in pathological corneal neovascularization [12,13]. The immature and incomplete blood vessels can increase blood vessel permeability, which causes chronic corneal edema, fat leakage, inflammatory reactions, and scarring [14]. The cornea with vascular distribution has a bad impact on vision and aesthetics. More seriously, it may lead to blindness, and people have a reduced prognosis for laser-assisted *in situ* keratomileusis, LASIK, penetrating keratoplasty, limbal autograft transplantation, and other procedures [15]. Therefore, the treatment of corneal neovascularization has become the focus of ophthalmological research [16]. In their review they discussed the pathophysiology, investigations, and various treatment options currently being undertaken as well as future therapeutic potentials. After corneal neovascularization occurs, the fat that leaks from the blood vessels into the cornea may cause secondary fatty keratopathy [17]. The fat may be deposited on the periphery of the cornea, near the center, or at the center, which will seriously affect the appearance of the eyes, vision, and eventually may cause blindness. At present, a variety of medical or surgical treatment methods have been developed, but the effects are limited with side effects. So the disease is a major challenge in ophthalmology.

The purpose of this study was to establish a New Zealand white rabbit animal model of fatty keratopathy, that is, the establishment of an animal model of fatty keratopathy. The goal was achieved by the combination of a corneal neovascularization animal model and a hyperlipidemia animal model. In addition, two groups were created according to the experimental sequence. The first group induced a pattern of corneal neovascularization followed by a pattern of hyperlipidemia, while the second group did the opposite. Since all major crystalline deposits of the cornea were apparent in both groups’ results, the animal models of fatty Keratopathy were successfully established in both groups.

## 2 Materials and methods

### 2.1 Experimental rabbit model of corneal neovascularization

A closed eye contact lens wear model was used to make this animal model. All the rabbits in the study were put under general anesthesia and had their eyes anesthetized for a week before being put on contact lenses and having the right eye haw removed. The rabbit’s right eye was fitted with a gas-impermeable contact lens (Boston PMMA gas-impermeable contact lens, 7.6 mm base curve, 13 mm diameter) one week later under general anesthesia and eye anesthetic, and an antibiotic eye ointment was used to prevent infection. To induce ocular hypoxia and neovascularization, the eyelid was briefly sutured with a 4-0 nylon suture. The temporary eyelid was removed after a week. Observations were recorded digitally, followed by the application of an antibiotic ointment and suturing of the eyelid again. All operations were performed by the same researcher to improve accuracy and consistency.


**Ethical approval:** The research related to animal use has been complied with all the relevant national regulations and institutional policies for the care and use of animals.

### 2.2 Experimental rabbit model of hyperlipidemia

The establishment of an animal model of hyperlipidemia was induced by feeding experimental rabbits with cholesterol-containing diets. The research team dissolved 10 g of cholesterol (C8503-5KG, Sigma-Aldrich) in 100 g of ordinary coconut oil, then added it to 900 g of rabbit dry feed and mixed them evenly, and the 1% cholesterol feed was configured. Nine experimental rabbits in this animal model were fed freely with cholesterol-added feed to induce hyperlipidemia. The same researcher had taken blood monthly to determine cholesterol and triglyceride concentrations in order to assess changes in blood lipids.

### 2.3 Experimental rabbit model of fatty keratopathy

It is expected that after combining corneal neovascularization, and the experimental rabbit model of hyperlipidemia, animal models of fatty keratopathy could be established. The animals were divided into two groups with two experimental rabbits in each group to compare the effect of the angiogenesis time point on fat deposition. The corneal neovascularization model was induced initially during the first four weeks, and the corneal neovascularization and hyperlipidemia models were induced simultaneously from the fifth week; the hyperlipidemia model was induced initially in the first week, and corneal neovascularization and hyperlipidemia models were induced simultaneously from the second week. The first group: the corneal neovascularization model was induced first in the first four weeks, and the corneal neovascularization and hyperlipidemia models were induced simultaneously from the fifth week; the second group: the hyperlipidemia model was induced first in the first week, and from the second week corneal neovascularization and hyperlipidemia models were induced. The operation was performed by the same researcher, and weekly observations and digital recordings were taken to evaluate the corneal neovascularization and fat distribution, which were compared by the same researcher in a single-blind experiment. After the model of fatty corneal keratopathy was established, all experimental rabbits were euthanized and the eyeballs were removed to examine histopathology.

## 3 Results

### 3.1 Experimental rabbit model of corneal neovascularization

After continuous recording and observation every week, it was found that in the first week, the corneal center had gradually produced a large range of sterile ulcers due to hypoxia and edema in and around the corneal ulcer, and that brush-shaped dense new blood vessels were generated outward from the eye wheel. Besides, the structure inside the eyeball was still clearly visible at this time. More severe corneal edema can be seen in the second week, and the new blood vessels in the eye wheel grow in a dendritic manner toward the center of the cornea. At this time, the new blood vessels began to converge with each other. Moreover, the structures inside the eyeball had begun to blur and could not be identified.

**Figure 1:**
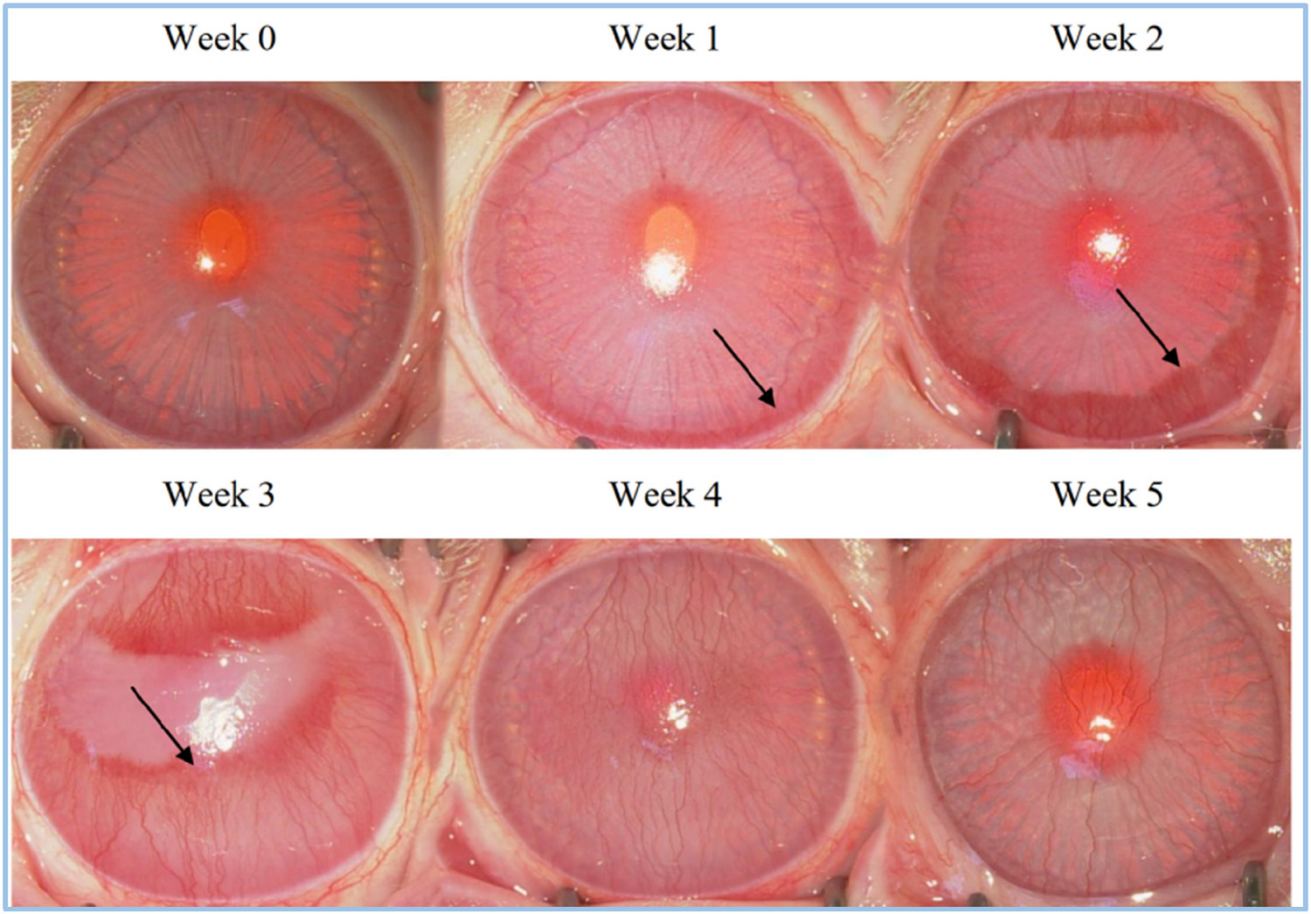
Observations of corneal neovascularization models.

In the third week, there was significant corneal edema, and neovascularization had entirely invaded the corneal core. The peripheral neovascularization at the center was more tightly coupled, and corneal edema and neovascularization almost completely covered the structure of the eyeball. As a consequence, the structure of the eye could not be identified. At about the fourth week, new blood vessels growing to the center of the cornea could be seen. The new blood vessels helped to improve the hypoxia in the center of the cornea and began to repair the cornea effectively. Therefore, corneal edema in some experimental rabbits was significantly improved (Figure 1). This animal model was easy to manipulate and was reproducible without significant eye complications.

### 3.2 Experimental rabbit model of hyperlipidemia

**Figure 2:**
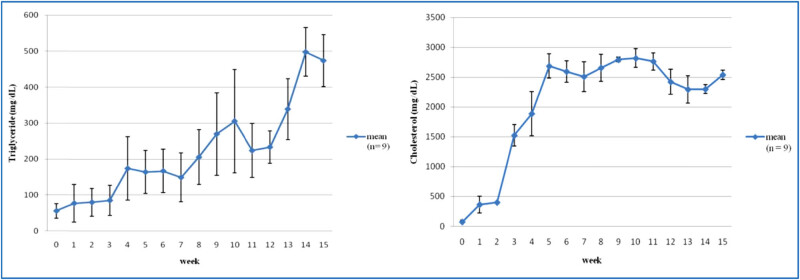
Changes in weekly blood triglyceride and cholesterol concentrations in hyperlipidemic animals.

Every week, blood samples were taken from rabbits to assess their blood lipids. The concentration of triglycerides in the blood has been observed to vary, and a gradual increase was observed. When rabbits were fed a high cholesterol diet in the fifth week, their average blood concentration was about 164.4 ± 59.5 mg/dL, which was three times the reference value range (39.2 ± 19.9 mg/dL). In contrast, in the first five weeks of high cholesterol diet, the blood cholesterol concentration would rise rapidly to a peak. The average blood concentration in the fifth week was about 2687.5 ± 201.26 mg/dL, which was 45 times of the reference value range (38.8 ± 20.6 mg/dL), with a small change after the fifth week, maintaining a balanced state (Figure 2). The reference values for triglyceride and cholesterol concentrations in blood were from Yu et al. [18]. This animal model effectively established an experimental rabbit model of hyperlipidemia.

### 3.3 Experimental rabbit model of fatty keratopathy-induced corneal neovascularization model

**Figure 3:**
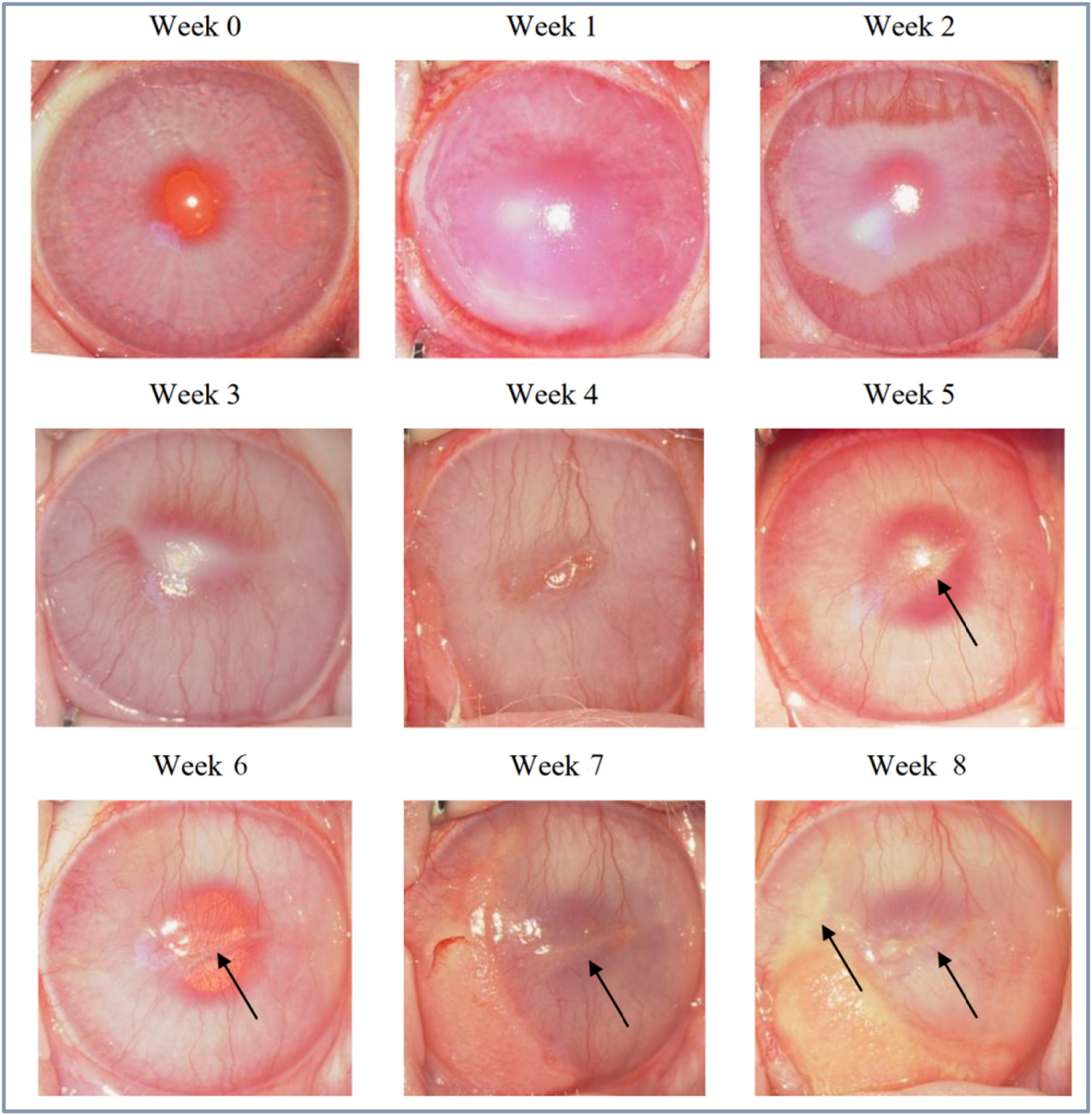
Results of the first group of animal models of fatty keratopathy.

**Figure 4:**
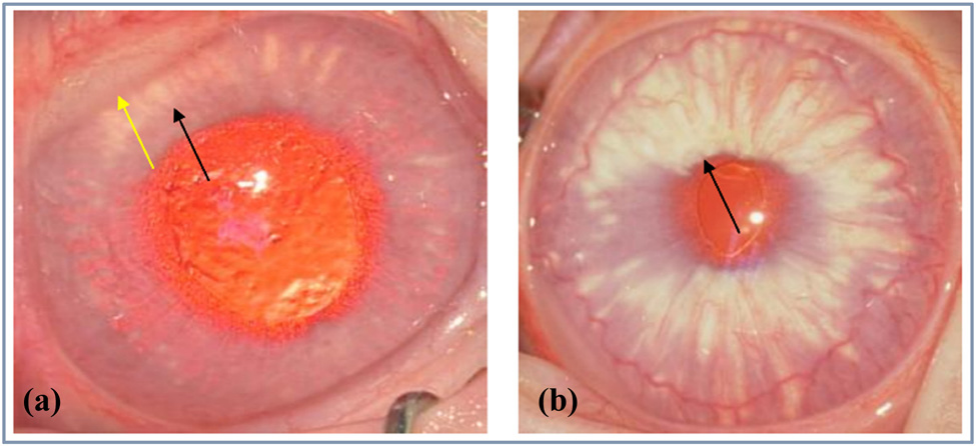
Results of fat deposition in normal eyes.

The first group induced corneal neovascularization and a model of hyperlipidemia at the same time in the fifth week, and the obvious yellow-white and crystal-reflective fat deposits at the center of the cornea were observed at week 5. Every week after that, the fat deposits became more pronounced and serious. The corneal center began to extend from the center toward the periphery. At the seventh to eighth week, granulation tissue was seen growing on the cornea of some experimental rabbits. Extensive fat deposition in the cornea was seen around the eighth week, which completely blocked the structure of the eyes and they could not be identified. In addition, after the granulation tissue subsided, the cornea covered by it showed relatively severe fat deposits (Figure 3). In the sixteenth week, the normal left eye of the experimental rabbit was found and infiltration and deposition of fat on the cornea near the eyeball and the iris was detected (Figure 4).

**Figure 5:**
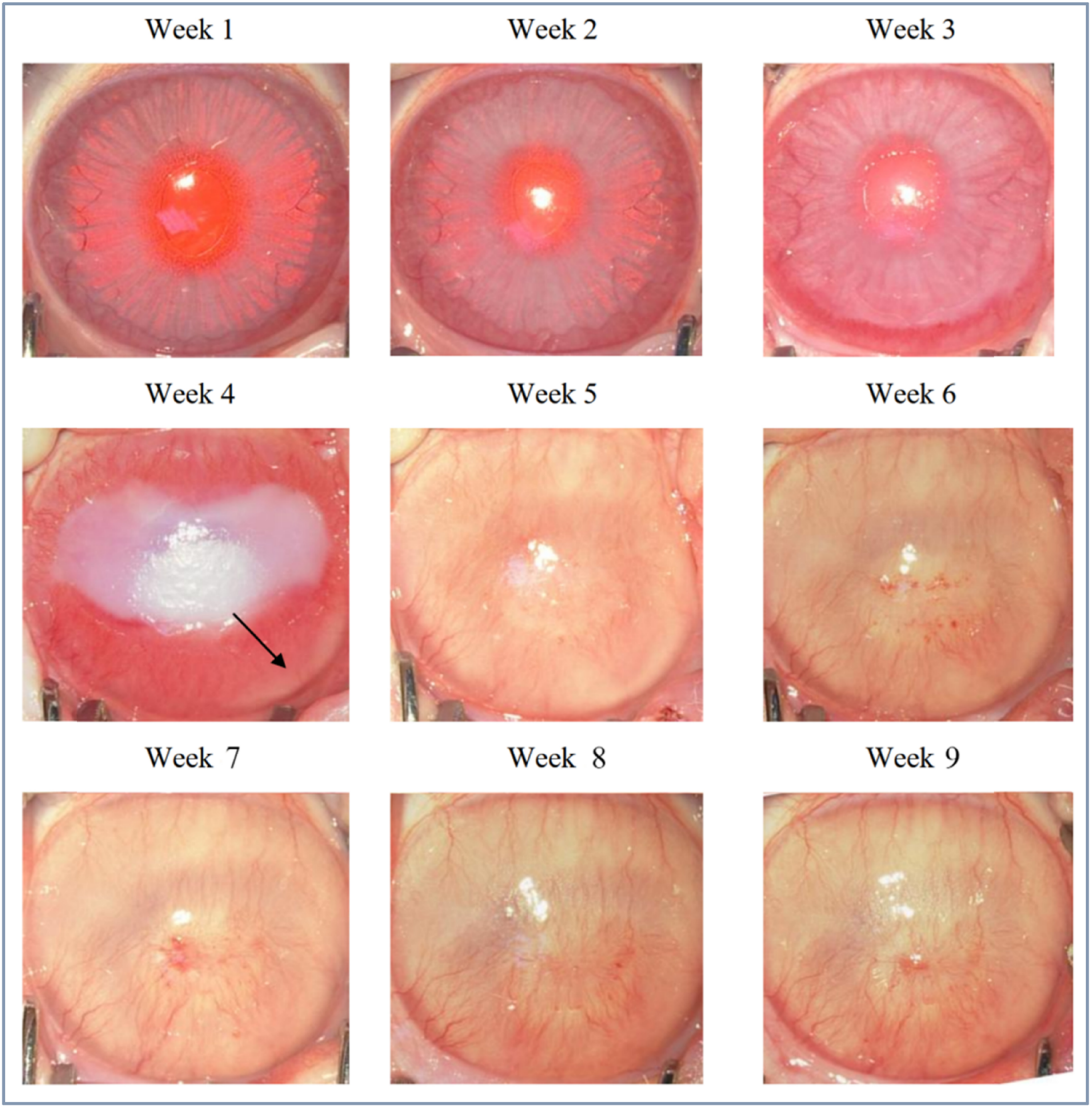
Results of the second group of animal models of fatty keratopathy.

The second group induced a hyperlipidemia model for one week and then induced a neovascularization and hyperlipidemia model in the second week. From the second to third week, new blood vessels started to grow from the eye wheel to the cornea. The yellow-white fat with crystal reflecting characteristics could be detected during the fourth week. It grew from the cornea’s perimeter, together with blood vessels, and gradually got deposited in the cornea’s core. A fairly extensive and diffuse fat deposit was visible at around the fifth week, which completely blocked the structure within the eye and therefore could not be identified (Figure 5). The infiltration and deposition of fat on the iris of the normal left eye of the experimental rabbit was found in the fifteenth week (Figure 4).

## 4 Discussion

Fat deposits were detected around the cornea and on the iris of normal eyes during an interventional rabbit model of hyperlipidemia. This was described in the experimental rabbit model of hyperlipidemia literature. Fat deposits near the cornea and around the cornea are similar to human arcus senilis, but the difference is in the way they are deposited. Peiffer et al., (2013) summarized the anatomy and physiology of the rabbit eye from a comparative perspective [19]. The experimental rabbit had almost complete deposition from the cornea to the eye wheel, and arcus senilis also had areas without fat deposition near the eye wheel. The yellow-white fatty plaques on the iris exist between the root of the iris and the pupil, which were initially small white spots that gradually merged to form a circular plaque [20]. Shah et al. also explained the translational preclinical pharmacologic disease models for ophthalmic drug development in their review [21]. In different animal model groups of fatty keratopathy, it was found that the first group induced the corneal neovascularization and hyperlipidemia mode after inducing the corneal hypoxia mode, and that the fat deposition will be mainly centered on the cornea, and then extends from the center to the periphery [21]. It was speculated that the main reason was that the new blood vessels have grown for more than two weeks, and more than 80% of them will have pericyte covering the blood vessels, which prevented the fat from easily penetrating, allowing it only to get deposited from the end of blood vessels [22]. In contrast, the second group, which had induced the hyperlipidemia model first, and then induced corneal neovascularization and hyperlipidemia model at the same time, experienced comprehensive corneal fat deposition. It was speculated that fat leakage began before the surrounding cells were covered when new blood vessels grew from the periphery of the cornea to the centre, and the peripheral cornea also exhibited significant fat deposition. One of the experimental rabbits that rejected the high cholesterol diets could only be induced to produce new blood vessels in the cornea, but not fatty keratopathy. This indicated that the fat deposition in the cornea and iris was related to the fat content in the blood. However, blood was not collected from this experimental rabbit for blood lipid testing. So it was not possible to know the blood fat content at that time. From the above observations, it was found that there is a significant correlation between fat leakage and blood fat content, and vascular permeability, while the permeability of new blood vessels is related to VEGF and therefore impedance VEGF can cope with the disease [23,24]. For example, bevacizumab application under the conjunctiva will be the focus of future research. In this experiment, an experimental rabbit model of hyperlipidemia was used, and in conjunction with corneal neovascularization animal models, animal models of fatty keratopathy were successfully derived. These two animal models are quite easy to be repeated and executed. Therefore, the stability and reproducibility of the animal models of fatty keratopathy are excellent. I hope the animal models will be helpful for future scholars to study ways to deal with fatty keratopathy.

## 5 Conclusion

The results of this experiment successfully induced excellent, stable, and highly reproducible animal models of fatty keratopathy, thereby providing future research opportunities in the treatment and control of the disease. The results from both the groups demonstrated significant crystalline deposits on the cornea and thus the animal models of fatty keratopathy were successfully established in both experimental groups.
